# Novel Random PBS-Based Copolymers Containing Aliphatic Side Chains for Sustainable Flexible Food Packaging

**DOI:** 10.3390/polym9120724

**Published:** 2017-12-16

**Authors:** Giulia Guidotti, Michelina Soccio, Valentina Siracusa, Massimo Gazzano, Elisabetta Salatelli, Andrea Munari, Nadia Lotti

**Affiliations:** 1 Civil, Chemical, Environmental and Materials Engineering Department, University of Bologna, Via Terracini 28, 40131 Bologna, Italy; giulia.guidotti9@unibo.it (G.G.); andrea.munari@unibo.it (A.M.); 2 Department of Chemical Science, University of Catania, Viale A. Doria 6, 95125 Catania, Italy; vsiracus@dmfci.unict.it; 3 Organic Synthesis and Photoreactivity Institute, ISOF-CNR, Via Gobetti 101, 40129 Bologna, Italy; massimo.gazzano@cnr.it; 4 Department of Industrial Chemistry “Toso Montanari”, University of Bologna, Viale del Risorgimento 4, 40136 Bologna, Italy; elisabetta.salatelli@unibo.it

**Keywords:** poly(butylene succinate), random copolymers, bio-based polyesters, thermal properties, mechanical properties, barrier properties, structure–property relationship

## Abstract

In the last decade, there has been an increased interest from the food packaging industry toward the development and application of biodegradable and biobased plastics, to contribute to the sustainable economy and to reduce the huge environmental problem afflicting the planet. In this framework, the present paper describes the synthesis of novel PBS (poly(butylene succinate))-based random copolymers with different composition containing glycol sub-units characterized by alkyl pendant groups of different length. The prepared samples were subjected to molecular, thermal, diffractometric and mechanical characterization. The barrier performances to O_2_, CO_2_ and N_2_ gases were also evaluated, envisioning for these new materials an application in food packaging. The presence of the side alkyl groups did not alter the thermal stability, whereas it significantly reduced the sample crystallinity degree, making these materials more flexible. The barrier properties were found to be worse than PBS; however, some of them were comparable to, or even better than, those of Low Density Polyethylene (LDPE), widely employed for flexible food packaging. The entity of variations in the final properties due to copolymerization were more modest in the case of the *co*-unit with short side methyl groups, which, when included in the PBS crystal lattice, causes a more modest decrement of crystallinity degree.

## 1. Introduction

Petroleum-based plastics are used in everyday life (their applications range in several different fields, such as packaging, building, automotive, electronics, agriculture, medicine, etc.), due to their low price, lightness, ease of production, ease of processability, modulation of properties and durability. This last characteristic, however, has caused over the years both huge terrestrial and marine pollution problems, due to accumulation of large quantities of plastic wastes in landfills or marine habitat. In fact, traditional fossil-based plastics, such as polyolefins, to cite one example, degrade very slowly, with consequent permanence for hundreds or even thousands of years in the environment [1,2].

Food packaging sector, which is the largest segment of application for plastics, is the one that contributes most to the production of plastic waste, the recovery and recycling of the end-of-life materials being very problematic, due to the contamination of package with food [3].

Nowadays, people as well as governments are more aware of the harmful effects of petrochemical derived plastic materials in the environment. Consequently, academic as well as industrial researchers have conducted many studies on managing plastic waste on earth by finding eco-friendly alternatives to traditional recalcitrant fossil-based plastics. A valid ecofriendly solution to this urgent need is represented by biodegradable plastics, which can be safely disposed in the environment, being able to easily degrade through the enzymatic actions of microorganisms. The degradation of biodegradable plastics indeed gives rise to carbon dioxide, methane, water, biomass, humic matter and various other natural substances, which can be readily eliminated.

Therefore, even though currently bioplastics represent only 1% of the about 320 million tons of plastic produced annually, their market is rapidly growing at about 20–100% per year. Bioplastics production is indeed expected to increase from around 4.2 million tons in 2016 to approximately 6.1 million tons in 2021 [4]. Such consistent increment is driven not only by the growing awareness of the impact of plastic materials on the environment, but also by the need to reduce their dependence on non-renewable fossil resources, which are subjected to price fluctuations and whose quantities are going to decrease in the near future.

In this context, biodegradable and bio-based plastics are the most preferable choice: bio-based poly(butylene succinate) (BioPBS), industrially produced by PTT MCC Biochem Company since 2016, belongs to this class, as it can be derived from natural resources, such as sugarcane cassava and corn, and is naturally compostable into biomass, carbon dioxide and water, without requiring a specialized composting facility. Moreover, it can be processed by employing the current extrusion coating machines, blown film extruders, and injection molding machines used for LDPE, with no additional investment; has excellent heat sealability; and has been approved for food contact.

The big interest in PBS and its copolymers has been proven by the large number of published works in the literature: different possible applications have been evaluated, such as mulching films, food packaging, tissue engineering and drug delivery; degradation kinetics studies in different environments have been also carried out [5,6,7,8,9,10,11,12,13,14,15,16,17,18,19,20,21,22,23,24,25,26,27,28,29,30,31]. Since 2009, our research group has given its contribution in this research topic, by synthesizing and studying several block and random copolymers of PBS: only recently, we investigated the barrier performances of PBS and some of its statistical copolymers [28,29,30,31].

As is well known, side alkyl groups, randomly distributed along the linear main chain, contribute to increase the material flexibility, reducing the crystallizing macromolecular chain ability [32,33]. Poly(butylene succinate) is too rigid for flexible food packaging, due to its high crystallinity degree: copolymerization with a glycol containing enough long side alkyl groups could represent an efficient tool to improve the mechanical properties. To the best of our knowledge, no random copolymers of PBS containing alkyl side groups have been synthesized and characterized yet from the point of view of molecular, thermal and mechanical properties, nor has barrier performance been evaluated to envisage the application of these materials in food packaging.

In this view, the final aim of the present paper is to establish structure–property relationship, with particular attention devoted to the effect of side group length on the material final properties.

## 2. Materials and Methods

### 2.1. Materials

Dimethyl succinate (DMS), 1,4-butanediol (BD), neopenthyl glycol (NG), 2-butyl-2-ethyl propanediol (BEPD) and titanium tetrabutoxide (Ti(OBu)_4_) were reagent grade products (Sigma-Aldrich, St. Louis, MO, USA); DMS, BD, NG and BEPD were used as supplied, whereas Ti(OBu)_4_ was distilled before use [34].

### 2.2. Polymer Synthesis

Poly(butylene succinate) (PBS) was synthesized from DMS and BD, while poly(butylene/2-butyl,2-ethyl-propylene succinate) (P(BS*_m_*BEPS*_n_*)) and poly(butylene/neopenthyl succinate) (P(BS_70_NS_30_)) random copolymers were prepared starting from different BD/BEPD and BD/NG ratios as glycol moiety, and DMS, as acid monomer. In all cases, 20% glycols molar excess was used with respect to diester content.

All the reactions were carried out in bulk, starting from 50 g of the monomers mixture and employing titanium tetrabutoxide as catalyst (about 150 ppm of Ti/g of polymer) in a 200 mL glass reactor, with a thermostated silicon oil bath; temperature and torque were continuously recorded during polymerization. The polymers were obtained according to the usual two-stage polymerization procedure [35,36]. In the first stage, under pure nitrogen flow, the temperature was set at 180 °C and kept constant until more than 90% of the theoretical amount of methanol was distilled off (about 120 min). In the second stage, the pressure was progressively reduced to 0.1 mbar to facilitate the removal of the glycol excess, and the temperature was raised to 230 °C. The syntheses were stopped after about four additional hours (up to a constant value of the measured Torque).

**Figure 1:**
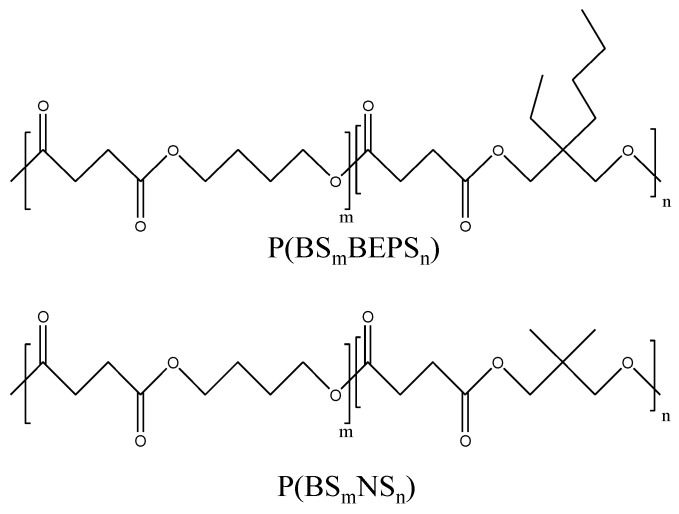
Chemical structures of P(BS*_m_*BEPS*_n_*) and P(BS*_m_*NS*_n_*) random copolymers.

Light yellow colored materials were obtained by using the described procedure. The molecular formula of PBS-based random copolymers is reported in Figure 1.

### 2.3. Film Preparation

Films were obtained by compression molding the polymer pieces between two Teflon plates (15 × 15 cm^2^), with an appropriate spacer, at a temperature *T* = *T*_m_ + 40 °C for 2 min under a pressure of 2 ton/m^2^ (Carver C12, laboratory press). Afterwards, the films have been cooled (≈65 °C·min^−1^) to room temperature keeping the applied pressure. Circular films of about 10 cm diameter and 100 μm thickness were obtained for each polymer.

Prior to the characterization, the films were stored under vacuum at room temperature for at least three weeks to reach the thermal equilibrium.

The film thickness was determined using a Digital Dial Indicator (MarCator 1086 type, Mahr GmbH, Esslingen, Germany), connected to a PC, using the Sample Thickness Tester DM-G software (Mahr GmbH, Esslingen, Germany). The reading was made measuring a minimum, a maximum and an average value. The results represent the mean value thickness of three experimental tests run at 10 different points on the polymer film surface at room temperature.

### 2.4. Molecular and Thermal Characterization

Molecular characterization was performed by means of proton and carbon nuclear magnetic resonance spectroscopy (^1^H-NMR and ^13^C-NMR) at room temperature, employing a Varian Inova 400-MHz instrument (Agilent, Technologies, Palo Alto, CA, USA). Operating settings: (i) ^1^H-NMR: relaxation delay of 0 s, acquisition time of 1 s and up to 100 repetitions and relaxation delay of 1 s; and (ii) ^13^C-NMR: acquisition time of 1 s, up to 700 repetitions and a full decoupling mode. The samples were dissolved in chloroform-d with 0.03 v % tetramethylsilane.

Molecular weights were evaluated by gel-permeation chromatography (GPC) at 30 °C using a 1100 HPLC system (Agilent Technologies, Santa Clara, CA, USA) equipped with PLgel 5-μm MiniMIX-C column (Agilent Technologies). A refractive index was employed as detector. Chloroform was used as eluent with a 0.3 mL/min flow and sample concentrations of about 2 mg/mL. A molecular weight calibration curve was obtained with polystyrene standards in the range of 2000–100,000 g/mol.

Thermogravimetric analysis (TGA) was carried out under nitrogen atmosphere using a Perkin Elmer (Waltham, MA, USA) TGA7 apparatus (gas flow: 40 mL/min) at 10 °C/min heating rate up to 900 °C.

Calorimetric measurements were conducted by using a Perkin Elmer DSC7 instrument. In the typical setup, the external block temperature control was set at −70 °C and weighed samples of ca. 10 mg were heated up to 40 °C above fusion temperature at a rate of 20 °C/min (first scan), held there for 5 min, and then rapidly quenched by immersing the pans in liquid nitrogen. Finally, they were reheated from −70 °C to a temperature well above the melting point of the sample at a heating rate of 20 °C/min (second scan). The glass-transition temperature (*T*_g_) was taken as the midpoint of the heat capacity increment Δ*c*_p_ associated with the glass-to-rubber transition. The cold crystallization temperature (*T*_cc_) and the melting temperature (*T*_m_) were determined as the peak value of the exothermal and endothermal phenomena in the DSC curve, respectively. The specific heat increment Δc_p_, associated with the glass transition of the amorphous phase, was calculated from the vertical distance between the two extrapolated baselines at the glass transition temperature. The heat of cold crystallization (Δ*H*_cc_) and the heat of fusion (Δ*H*_m_) of the crystalline phase were calculated from the total areas of the DSC exotherm and endotherm, respectively.

### 2.5. Wide-Angle X-ray Analysis

X-ray diffraction patterns were obtained with CuKα radiation in reflection mode by means of an X’Pert PANalytical diffractometer (PANalytical, Almelo, The Netherlands) equipped with a fast X’Celerator detector, 0.1° step, 100 s/step. The samples were analysed in form of films. The indices of crystallinity (Xc) were calculated from the X-ray diffraction profiles by the ratio between the crystalline diffraction area (*A*_c_) and the total area of the diffraction profile (*A*_t_), X_c_ = *A*_c_/*A*_t_. The crystalline diffraction area was obtained from the total area of the diffraction profile by subtracting the amorphous halo. The incoherent scattering was taken into consideration. The unit cell parameters were calculated by whole pattern fitting using Powder Cell 2.3 for Windows [37].

### 2.6. Stress–Strain Measurements

The tensile testing of PBS and its random copolymers was performed using a Zwick Roell Texture machine (Ulm, Germany), equipped with rubber grip and controlled by computer. A pre-load of 1 MPa with a 5 mm/min speed, on a 500 N load cell, was used. Films (5 × 50 mm^2^) with an initial grip separation of 23 mm were employed. The stress–strain measurements were performed with a crosshead speed of 50 mm/min. Five different samples from the same film were tested for each copolymer composition and the results were provided as the average value ± standard deviation. All tests were carried out in accordance with ASTM D638 procedure.

### 2.7. Gas Transport Measurements

The determination of the gas barrier behavior was performed by a manometric method using a Permeance Testing Device, type GDP-C (Brugger Feinmechanik, GmbH, München, Germany), according to ASTM 1434-82 (Standard test Method for Determining Gas Permeability Characteristics of Plastic Film and Sheeting), DIN 53 536 in compliance with ISO/DIS 15 105-1 and according to Gas Permeability Testing Manual (Registergericht München HRB 77020, Brugger Feinmechanik GmbH).

After a preliminary high vacuum desorption of the up and lower analysis chambers, the upper chamber was filled with the gas test, at ambient pressure. A pressure transducer, set in the lower chamber, records continuously the increasing of gas pressure as a function of the time [38,39]. The gas transmission rate (GTR) was determined considering the increase in pressure in relation to the time and the volume of the device. All the measurements have been carried out at room temperature of 23 °C. The operative conditions were: gas stream of 100 cm^3^·min^−1^; 0% RH of gas test, food grade; sample area of 78.5 cm^2^ (standard measurement area). Gas transmission measurements were performed at least in triplicate and the mean value is presented.

## 3. Results and Discussion

### 3.1. Molecular Characterization

The molecular characterization data of the polymers under investigation are reported in Table 1: all the samples were characterized by high and similar molecular weights, proving that no significant thermal degradation reactions occurred during the polymerization.

**Figure 2:**
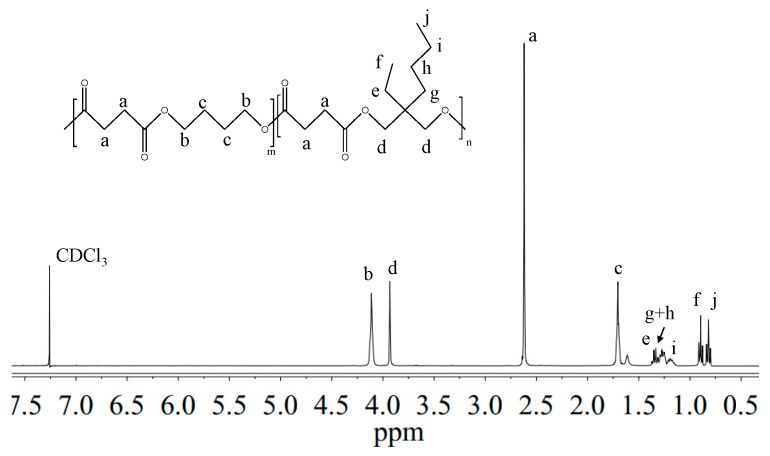
^1^H-NMR spectrum of P(BS_70_BEPS_30_) with resonance assignments.

^1^H-NMR and ^13^C-NMR analysis have been carried out to: (i) verify the chemical structure; (ii) calculate the actual composition; and (iii) calculate the degree of randomness (b). As an example, Figure 2 reports the ^1^H-NMR spectrum of P(BS_70_BEPS_30_) with the corresponding resonance assignments.

**Figure 3:**
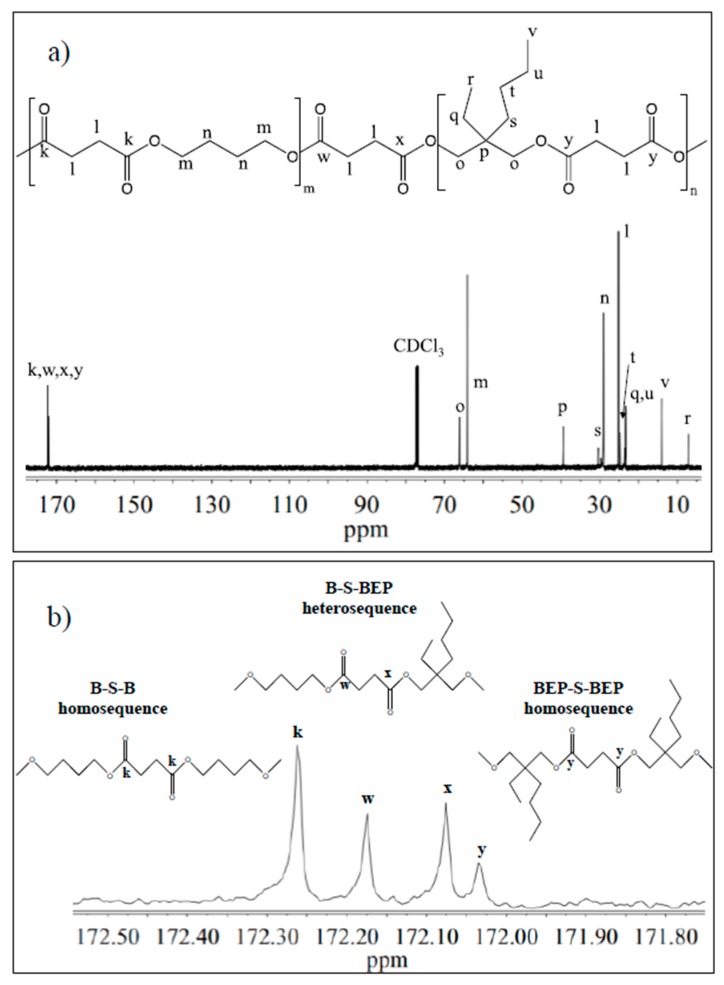
(**a**) ^13^C-NMR spectrum of P(BS_70_BEPS_30_) with resonance assignments; and (**b**) enlargement of ^13^C-NMR spectrum in the region 172.50–171.80 ppm together with the schematic representation of B-S-B, B-S-BEP and BEP-S-BEP sequences.

The chemical structure of the copolymer is confirmed, since no additional peaks were found in the spectrum. The copolymer composition was determined from the relative areas of the resonance peak of the *b* protons of the butylene sub-unit, located at 4.11 ppm and of the signal at 3.90 ppm corresponding to the *d* protons of the butyl-ethyl propylene moiety (see Figure 2). For all the copolymers, the actual composition is close to the feed one (see Table 1), proving a good control in the polymerization process. The degree of randomness (b) has been calculated by ^13^C-NMR spectroscopy. Figure 3 reports the ^13^C-NMR spectrum of P(BS_70_BEPS_30_) with the peaks assignments (Figure 3a) and the magnification of the region in between 172.50 and 171.80 ppm, where the signals due to the ester groups carbons are located (Figure 3b). In this region, together with the signals of the ester carbons at 172.26 and 172.03 ppm, corresponding to the B-S-B (*k* carbon) and BEP-S-BEP (*y* carbon) homosequences, respectively, two additional peaks in between can be detected. These signals are due to the B-S-BEP and BEP-S-B (*w* and *x* carbons) heterosequences, due to transesterification reactions. The degree of randomness b has been calculated from the intensity of the k, y, w and x peaks.

It is worth noting that *b* is equal to 1 for random copolymers, equal to 2 for alternate copolymers, equal to 0 for a mixture of two corresponding homopolymers and 0 < *b* < 1 for block copolymers. The degree of randomness was calculated according to Equation (1):

(1) $$b=P_{B-BEP}+P_{BEP-B}$$

where *P*_B-BEP_ and *P*_BEP-B_ are the probability of finding a B unit next to a BEP one and the probability of finding a BEP unit close to a B one, respectively.

In turn, the two probabilities can be expressed as follows (Equation (2)):

(2) $$P_{B-BEP}=\frac{I_{w}}{I_{w}+I_{k}};\;P_{BEP-B}=\frac{I_{x}}{I_{x}+I_{y}}$$

where, *I_k_*, *I_w_*, *I_x_*, and *I_y_* represent the integrated intensities of the resonance peaks of the B-S-B, B-S-BEP, BEP-S-B and BEP-S-BEP sequences, respectively (Figure 3b).

For all copolymers, the calculated b equals 1. Therefore, we can conclude that the experimental conditions adopted allowed us to synthesized copolymers with random distribution of sequences.

### 3.2. Thermal and Structural Characterization

The thermal stability of the samples under investigation has been analyzed by TGA under nitrogen flow. The temperatures of peak onset (*T*_onset_) and of the maximum weight loss rate (*T*_max_) have been collected in Table 2. In all cases, the weight loss takes place in one-step and is 100% (TGA curves not shown). All copolymers under study show very good thermal stability, the weight loss starting above 380 °C, similar to that of PBS homopolymer. Copolymerization therefore does not affect PBS thermal stability, *T*_onset_ and *T*_max_ being almost constant in the whole composition range studied.

**Figure 4:**
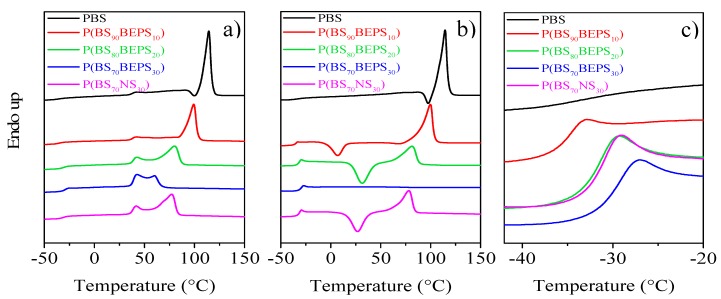
Calorimetric curves (heating rate: 20 °C/min) of PBS, P(BS_m_BEPS_n_) and P(BS_70_BEPS_30_) copolymers: (**a**) 1st scan; (**b**) 2nd scan after quenching from the melt; and (**c**) magnification in the *T*_g_ region of 2nd scan.

The calorimetric curves and the relative thermal data of the samples under investigation are reported in Figure 4 and Table 2, respectively. As regards calorimetric study, we can exclude an influence of molecular weight on the glass transition and melting of the polymers synthesized, being the samples characterized by high and similar *M*_n_.

As one can see from the first calorimetric scan (Figure 4a), all the samples are semi-crystalline, the corresponding DSC traces being characterized by the presence of a baseline deviation around –30 °C, associated to the glass to rubber transition, followed by an endothermic peak at higher temperature, related to the melting of the crystalline portion. However, some differences can be evidenced, mainly corresponding to the melting peak, which progressively moves towards lower temperature and decreases in intensity (Δ*H*_m_ values lower) as the *co*-units amount increases, probably due to the formation of a crystalline phase with lower degree of perfection. This phenomenon is also accompanied by the increasing of the amorphous fraction, as indicated by the increased glass transition step height (Δ*c*_p_ values increase). Furthermore, the copolymers containing higher amount of comonomer, i.e., 20 and 30 mol % of BEPS and 30 mol % of NS *co*-units, show multiple melting peaks. This shape is typical of melting/recrystallization processes of low perfection crystals that usually develop in copolymeric systems, due to the hindering effect of the *co*-units in the crystallization process [40,41,42,43].

**Figure 5:**
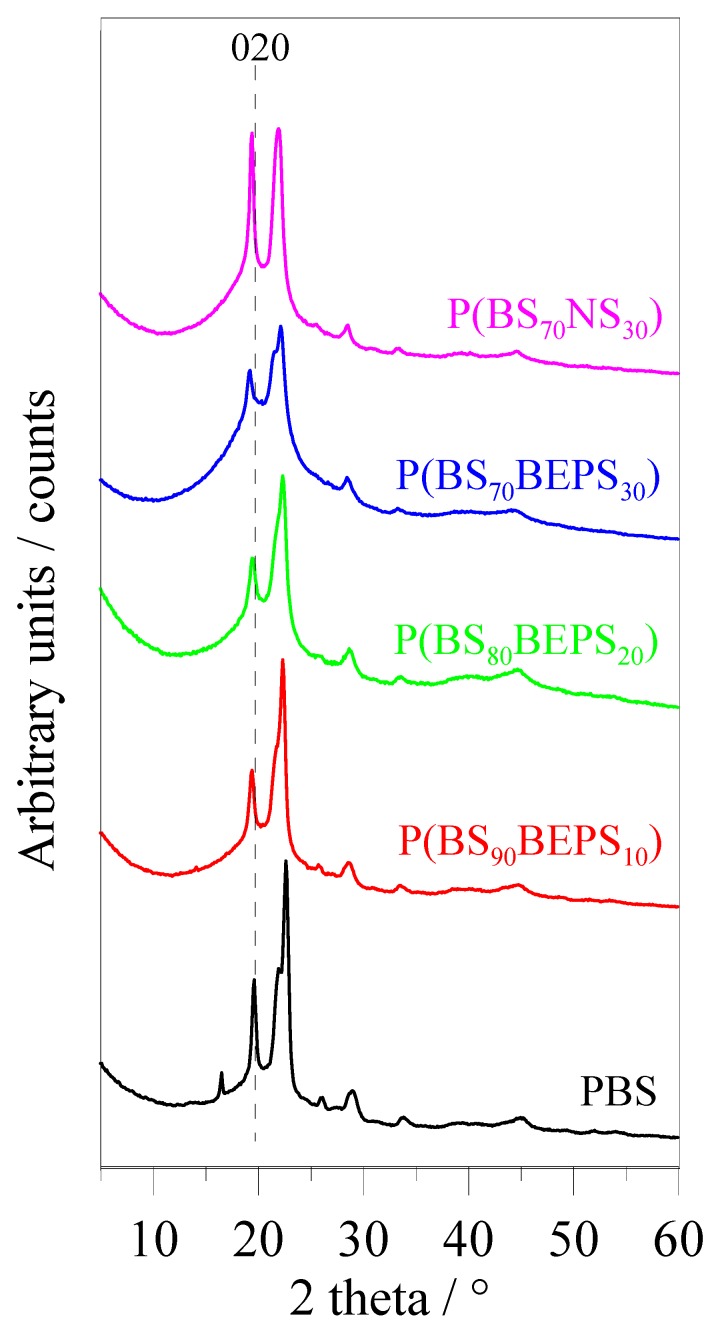
XRD profiles for the polymers under study. For PBS, the *0 2 0* reflection is indicated.

To investigate the crystal structure present in the random copolymers under investigation, X-ray diffraction (XRD) measurements were carried out. The patterns obtained are reported in Figure 5.

As one can see, all samples show the peaks characteristic of the α-PBS crystal phase [44]. The observed progressive increase of the area under the bell shaped background line as BEPS *co*-units fraction rises is due to the relative decrease of crystalline portion and consequent relative growth of the amorphous one in the copolymers, as also confirmed by the decrement of crystallinity degree values (X_c_) (see Table 3). In addition, along the series, the position of *0 2 0* reflection shifted to smaller 2-theta angles, i.e., to bigger interlayer distances.

For each sample, the parameters of the unit cell have been calculated and reported in Table 3.

An increase of the unit cell volume has been observed for P(BS*_m_*BEPS*_n_*): anyway, this, although detectable, is slight and not compatible with the inclusion of the BEPS segments in the α-PBS crystal cell. It has to be emphasized that the distortions of the crystal lattice (e.g., increase of the d-spacings) can be attributed partially to comonomer inclusion. Crowding effects of excluded *co*-monomers at the surface of the crystal may indeed also contribute to changes in the crystal lattice parameters. As reported for other systems previously investigated by us [23,29], the volume expansion in P(BS*_m_*BEPS*_n_*) samples has to be considered as a consequence of the disorder induced by the presence of comonomeric units, also confirmed by the reflections broadening (see Full Width High Medium measurement of *0 2 0* peak in Table 3). In this view, the complete exclusion of the BEPS *co*-units from the PBS crystal lattice can be hypothesized.

It is interesting to note the different effect of the two *co*-units, BEPS and NS, on the crystal phase developed in the corresponding copolymers, i.e., P(BS_70_BEPS_30_) and P(BS_70_NS_30_). The crystallization capability of the NS-containing copolymer is higher than that of BEPS-based one, being the X_c_ values equal to 31 and 18%, respectively. Moreover, the peak broadening (see FWHM column in Table 3), usually taken as a draft index of the overall order degree, has been found equal for PBS and P(BS_70_NS_30_), whereas the one of P(BS_70_BEPS_30_) is around 30% higher. On the other side, as reported in Table 3, the cell volume expansion occurring in P(BS_70_NS_30_) is only slightly bigger than the one detected for P(BS_70_BEPS_30_), even though appeared to be +20 *Å^3^* respect to PBS. Such value is the volume occupancy empirically estimated for one atom different from hydrogen in the crystal cell [45]. This result might be explained as due to the inclusion in the PBS unit cell of NS unit: in the presence of methyl substituents as in NS moiety, the unit cell can expand and statistically host them. The increase of the *a* and *b* parameters, *c* remaining constant, is coherent with the structure of α-PBS crystal phase because the polymeric chain is oriented in the *c*-axis direction and indeed insertion of NS-units does not provoke a chain extension, but rather a chain thickening.

In conclusion, although in both copolymeric systems investigated a similar lattice distortion is detected, its origin is different: disorder due to longer substituents for P(BS*_m_*BEPS*_n_*) versus possible inclusion of the short ramified units in P(BS*_m_*NS*_n_*).

As is well known, a partially crystalline material usually exhibits different glass transition behavior than the completely amorphous analog. In fact, although some conflicting results are reported in the literature [46], crystallinity usually acts like crosslinking and raises *T*_g_ values through its restrictive effect on the segmental motion of amorphous polymer chains. Therefore, to study the influence of chemical structure on the glass transition of random copolymers, the phenomenon should be examined in the total absence of crystallinity. In this view, all the samples under investigation were subjected to rapid cooling (quenching) from the melt (see Experimental Section for the details). The DSC curves and the thermal characterization data of the so-treated samples are reported in Figure 4b and collected in Table 2 as a function of *co*-units content.

The effect of copolymerization on the thermal behavior of PBS is evidenced by the calorimetric curves obtained after melt quenching. As one can see, while the PBS curves of the I and II scans are practically the same, evidencing the high crystallization rate of this homopolymer, that cannot be quenched in the amorphous state under the experimental conditions adopted, the DSC traces of all the copolymers drastically change after melt quenching in liquid nitrogen. Firstly, the glass transition phenomenon becomes more evident, as a consequence of an increased amorphous phase amount in the quenched samples. Secondly, a different crystallization capability can be evidenced, as the BEPS *co*-units content is increased. In particular, for the samples containing up to 20 mol % of BEPS *co*-units, the corresponding DSC traces are characterized by the glass to rubber transition step, an exothermic peak and an endothermic one, located at higher temperatures. This kind of DSC trace is typical of materials that, once the glass transition temperature is exceeded, can crystallize in the temperature window between *T*_g_ and *T*_m_ and then undergo melting of the crystals present in the specimen. Nevertheless, composition affects the area under the exothermic (Δ*H*_cc_) and the endothermic (Δ*H*_m_) peaks. For P(BS_90_BEPS_10_), the calorimetric results evidence the copolymer chains have not been locked in the disordered phase, since the amount of melting crystals is higher than the quantity of crystalline portion formed at T_cc_ (ΔH_cc_ < Δ*H*_m_). For P(BS_80_BEPS_20_) instead, being the areas under the two peaks equal (Δ*H*_cc_ = Δ*H*_m_), we can assert that the copolymer had been quenched into the amorphous phase, the presence of BEPS *co*-units along the PBS chains hindering its crystallization capability. This effect is even more evident in the copolymer containing the highest BEPS *co*-unit content, (P(BS_70_BEPS_30_)), for which just the glass to rubber transition step has been detected in the second scan DSC trace.

The DSC curve of the NS containing copolymer, P(BS_70_NS_30_), appeared to be very similar to the one of P(BS_80_BEPS_20_), despite their different molar composition: both samples are indeed able to crystallize during the II heating scan. This result, in line with the diffractometric analysis, confirms the higher hindering effect on PBS crystallization capability of the long side chains present in BEPS *co*-units with respect to the NS short pendants.

The *co*-unit nature has a direct effect also on the polymer chain mobility as indicated by the *T*_g_ and the corresponding Δc_p_ values reported in Table 2, and as shown by the DSC curves magnification of Figure 4c. As already reported in literature, the presence of alkyl pendant groups hinders the rotation around the C–C σ bond, because of their high steric hindrance, reducing the macromolecular mobility (*T*_g_ rises). However, if the pendant groups are long enough, they can exert an internal plasticizing effect, which leads to a decrement of the *T*_g_ value. The entity of the effect is proportional to the length of the side alkyl group. In the copolymers under study, both these opposite effects are supposed to influence chain mobility, i.e., *T*_g_ position.

To analyze the effect of the presence of the BEPS and NS *co*-units on the *T*_g_ of PBS homopolymer, in Figure 4c, we have reported the temperature region at which the glass to rubber transition occurs.

As one can see, the *T*_g_ step, rather low for the semi-crystalline PBS, increases in height in the copolymers, as a consequence of the increased amorphous phase portion. In particular, for the high BEPS *co*-units content copolymers, that have been quenched in the totally amorphous state, any restriction effect related to the presence of crystalline netpoints, leading to *T*_g_ increment, can be excluded and therefore the *T*_g_ position is due solely to chain mobility. It is interesting to note that, for these samples, despite the absence of crystalline domains, the glass to rubber transition step progressively moves towards higher temperature (see also Table 2), similarly to other copolymeric systems previously investigated [35,36]. In the case of P(BS_70_BEPS_30_), the increment in the value of glass transition temperature with respect to the one of completely amorphous PBS, previously determined [29], is 10 °C. This result seems to suggest that, for P(BS*_m_*BEPS*_n_*) copolymers, the steric hindrance of the alkyl side pendant prevails on their plasticizing effect. For the NS *co*-unit containing copolymer, P(BS_70_NS_30_), the balance of the steric hindrance and plasticizing effect of the side pendants is different, and determine the same *T*_g_ increase with lower *co*-unit weight fraction. In fact, as one can see in Figure 4c, the calorimetric trace of P(BS_70_NS_30_), whose *co*-unit weight fraction corresponds to 30 wt %, is practically overlapped with that of P(BS_70_BEPS_30_), whose *co*-unit weight fraction is 41 wt % (see Table 1). This result evidences that in the case of NS comonomeric units, the internal plasticizing effect is minor, probably due to the lower length of the neopenthyl side chains.

### 3.3. Mechanical Characterization

**Figure 6:**
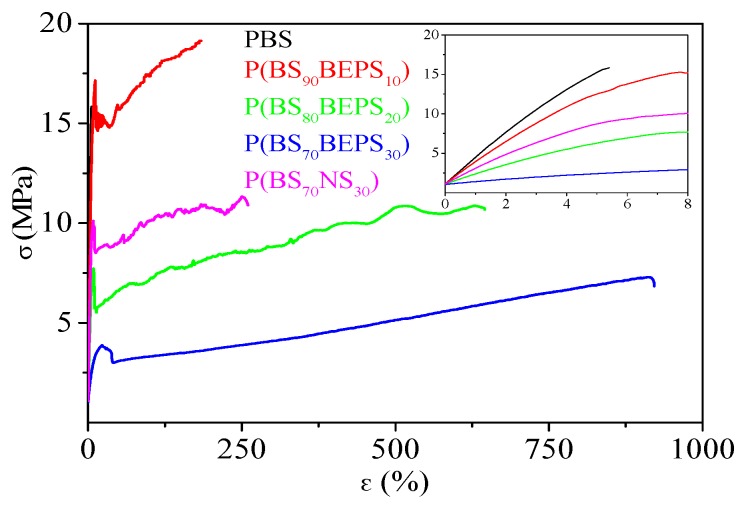
Stress–strain curves of PBS, P(BS*_m_*BEPS*_n_*) and P(BS_70_NS_30_) random copolymers; in the inset: magnification of the low σ vs. ε region.

To test the suitability of the materials synthesized for the intended application, tensile measurements have been performed. The stress–strain curves are shown in Figure 6 and the mechanical data (elastic modulus *E*, stress at break σ_b_ and elongation at break ε_b_) are listed in Table 4.

PBS homopolymer appeared to be the most rigid material among the investigated ones, being characterized by the highest elastic modulus and stress at break, accompanied by the lowest elongation at break. The introduction of BEPS *co*-units affects the mechanical response of PBS homopolymer, causing the decrease of both E and σ_b_. The entity of variation is function of copolymer composition: the higher the *co*-unit amount, the larger the effect. This result is probably related to the gradual reduction of the crystallinity degree, X_c_, in the copolymers with respect to the homopolymer. In particular, for the highest BEPS *co*-units content, E decreases by almost one order of magnitude, while σ_b_ is halved. The presence of BEPS comonomeric units also leads to a smart increment of ε_b_, from 5% for PBS up to 1050% for P(BS_70_BEPS_30_), this copolymer behaving as an elastomer.

Nevertheless, the crystallinity degree is not the only parameter determining the mechanical properties of the random copolymers under study. The effect of the different pendant group length, already evidenced by calorimetric measurements, is also responsible for a different mechanical behavior. In fact, if we consider P(BS_80_BEPS_20_) and P(BS_70_NS_30_), whose X_c_ values are very similar, the copolymer containing BEPS sequences presents lower E and σ_b_ together with a higher ε_b_. This trend, not explainable based on the degree of crystallinity, which is the same for both samples, has to be ascribed to the higher chain flexibility and plasticizing effect imparted by the longer pendant alkyl chain present in the BEP glycol sub-unit.

### 3.4. Gas Barrier Performances

Gas barrier performances to dry N_2_, O_2_ and CO_2_ gases were evaluated at 23 °C, as such molecules are the main gases used for food packaging application, especially for Modified Atmosphere Packaging technique (MAP), with the aim of delaying senescence and increasing the shelf-life of packaged fresh food [28,47,48]. As reported by Caleb et al., O_2_ gas is responsible for the food respiration rate and a decrease in food respiration rate could delay the enzymatic degradation, extending the shelf- life of the packed food. However, if the O_2_ level is too low, tissue deterioration could occur, leading off-flowers and off-odors production [48]. CO_2_ gas confers a significant level of antimicrobial behavior on the food packaged, well explained by Farber [49] in his review. N_2_ is an inert gas used to complete the 100% of inside package atmosphere, preventing also the film collapse. It is insoluble in water and does not react with the food.

**Figure 7:**
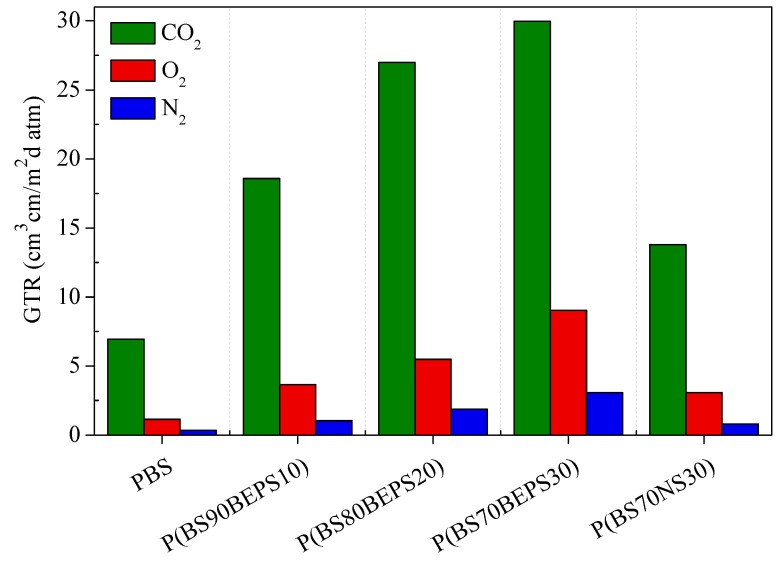
GTR of N_2_, O_2_ and CO_2_ through PBS, P(BS*_m_*BEPS*_n_*) and P(BS_70_NS_30_) films, at 23 °C.

The permeability values, expressed as Gas Transmission Rate (GTR), are collected in Table 5 and shown in Figure 7 for the films under analysis.

It can be seen that, in all cases, the N_2_-GTR shows the lowest values, whereas the CO_2_-GTR the highest ones, despite its greater molecular diameter (3.4 Å) with respect to nitrogen molecule (2.0 Å) molecules. The O_2_-GTR values lie in between. The values measured indicate that the gas permeability of all permeants through the copolymer film is higher than that of PBS film, highlighting that the introduction of aliphatic pendant chains influences the final barrier properties. The observed trend can be explained based on a reduction in sample crystallinity degree: in fact, as is well known, gas molecules diffuse and permeate more easily through the amorphous region, where the polymer chain mobility for these samples is particularly enhanced, as evidenced by the low *T*_g_ values, well below room temperature [51].

The length of alkyl pendant groups influences the barrier behavior too: from the comparison between P(BS_70_NS_30_) and P(BS_70_BEPS_30_), which have very similar composition, one can see that the former has superior barrier performances (the GTR values of P(BS_70_NS_30_) are almost halved), which can be ascribed to its higher crystallinity degree. Interestingly, P(BS_70_NS_30_) barrier performances are even better with respect to those of P(BS_80_BEPS_20_), despite the very similar crystallinity degree for the two copolymers. The observed result can be explained taking into account that the shorter aliphatic pendant chain (methyl groups) stiffens the macromolecular main chain more significantly and that NS *co*-units entering in crystal lattice of PBS render the crystal phase more packed. In conclusion, P(BS_70_NS_30_) copolymer has the best barrier performances with respect to N_2_ and CO_2_ among the copolymers investigated and GTR to oxygen comparable to that of P(BS_90_BEPS_10_).

Low Density Polyethylene (LDPE) dominates the market of food and beverage flexible package and is characterized by high degree of short-chain branching and long-chain branching. The GTR values to oxygen and carbon dioxide for this traditional fossil-based plastic have been added in Table 5 for the sake of comparison. Both PBS homopolymer and P(BS_70_NS_30_) copolymer displayed superior barrier properties with respect to LDPE to both gases, whereas P(BS_90_BEPS_10_) showed comparable barrier behavior. By increasing the BEPS-unit percentage, the permeability performances worsened, even though not dramatically, confirming that the modification of the chemical structure influences the final barrier behavior.

Even though this comparison is far from exhaustive, it provides important evidence of the good potentiality of the copolymers under investigation to be used for eco-friendly flexible food packaging application.

## 4. Conclusions

Novel high molecular weight PBS-based random copolymers containing different amounts of glycol sub-units with pendant groups of different length were successfully synthesized through an eco-friendly solvent-free process. Comonomeric units did not alter thermal stability, a crucial factor during material processing. Long pendant groups reduce significantly the PBS crystallizing ability: a drastic reduction of crystallinity degree is indeed observed. Such effect is not so marked with the shortest methyl side groups, which are included in the PBS crystal lattice.

The long alkyl pendant groups significantly affect the mechanical properties, reducing and increasing the elastic modulus and elongation at break, respectively. The introduction of 30 mol % of BEPS *co*-units along PBS polymer chains confers to the final material an elastomeric behavior.

Gas permeability properties can be nicely tailored, acting both on pendant group length and on copolymer composition, in view of the desired packaging application. In particular, a decrease in the barrier performances with the increase of the BEPS *co*-unit content was observed, due to a decrease of crystallinity degree. The increment of GTR values were more consistent with CO_2_ gas test, whereas more modest with N_2_ one. Concerning the O_2_-GTR value variations, these lie in between. Such results are of fundamental importance for MAP technology, in order to select the suitable headspace gas composition. An atmosphere poor in O_2_ and rich in CO_2_ slows down the metabolism of packed products or the spoilage activity, maintaining or prolonging the desired product shelf-life. On the other hand, N_2_ gas enables the formation of an inert atmosphere preventing the packaging collapse. Consequently, it is important that its internal percentage keeps constant. Lastly, the barrier performances of some copolymers appeared to be comparable or even superior with respect to those of LDPE, widely employed in flexible food packaging.

## Figures and Tables

**Table 1:** Molecular characterization data of PBS and P(BS*_m_*BEPS*_n_*) and P(BS_70_NS_30_) random copolymers.

Samples	BS (mol %) Feed	BS (mol %) by ^1^H-NMR	BS (wt %)	*M*_n_ (g/mol)	D
PBS	100	100	100	50,000	2
P(BS_90_BEPS_10_)	90	87	83	52,000	1.9
P(BS_80_BEPS_20_)	80	78	72	49,000	2.1
P(BS_70_BEPS_30_)	70	67	59	63,000	2.2
P(BS_70_NS_30_)	70	72	70	55,000	2.1

**Table 2:** Thermal characterization data of PBS and P(BS*_m_*BEPS*_n_*) and P(BS_70_NS_30_) random copolymers.

Samples	*T*_onset_ °C	*T*_max_ °C	I SCAN				II SCAN					
			*T*_g_ °C	Δ*_C_*_p_ J/g·°C	*T*_m_ °C	Δ*H*_m_ J/g	*T*_g_ °C	Δ_Cp_ J/g°C	*T*_cc_ °C	Δ*H*_cc_ J/g	*T*_m_ °C	Δ*H*_m_ J/g
PBS	385	407	−35	0.088	114	50	−35	0.192	-	-	114	51
P(BS_90_BEPS_10_)	381	410	−33	0.155	99	38	−34	0.180	7	19	100	50
P(BS_80_BEPS_20_)	382	416	−29	0.263	42 80	33	−31	0.444	31	34	81	34
P(BS_70_BEPS_30_)	382	413	−28	0.354	43 60	22	−29	0.457	-	-	-	-
P(BS_70_NS_30_)	386	415	−28	0.244	42 77	33	−29	0.456	27	30	79	30

**Table 3:** Crystallinity index (X_c_), width of the *0 2 0* peak (FWHM) and unit cell parameters.

Samples	X_c_ %	FWHM °	*a Å*	*b Å*	*c Å*	β °	V *Å^3^*
α-PBS *	-	-	5.23	9.12	10.90	123.9	431.5
PBS	46	0.43	5.24	9.09	10.85	123.7	429.9
P(BS_90_BEPS_10_)	38	0.54	5.29	9.19	10.72	123.5	434.6
P(BS_80_BEPS_20_)	31	0.66	5.27	9.22	10.70	123.3	437.7
P(BS_70_BEPS_30_)	18	0.55	5.29	9.39	10.70	122.9	446.1
P(BS_70_NS_30_)	31	0.43	5.31	9.26	10.87	122.2	451.3

* From Ref. [44].

**Table 4:** Mechanical characterization data of PBS and its random copolymers.

Samples	*E* (MPa)	σ_b_ (MPa)	ε_b_ (%)
PBS	301 ± 25	16 ± 2	5 ± 1
P(BS_90_BEPS_10_)	282 ± 17	17 ± 2	166 ± 26
P(BS_80_BEPS_20_)	139 ± 7	11 ± 1	675 ± 27
P(BS_70_BEPS_30_)	47 ± 7	7 ± 1	1050 ± 99
P(BS_70_NS_30_)	219 ± 12	18 ± 1.0	340 ± 34

**Table 5:** Gas transmission rate (GTR) data for PBS, P(BS*_m_*BEPS*_n_*) and P(BS_70_NS_30_) random copolymers, normalized for the thickness film sample, at 23 °C, with N_2_, O_2_ and CO_2_ dry gas test.

Sample	Film Thickness µm	N_2_-GTR cm^3^·cm/m^2^·d·atm	O_2_-GTR cm^3^·cm/m^2^·d·atm	CO_2_-GTR cm^3^·cm/m^2^·d·atm
PBS	112 ± 11	0.3473	1.1327	6.8573
P(BS_90_BEPS_10_)	102 ± 14	1.0286	3.6146	18.3380
P(BS_80_BEPS_20_)	109 ± 15	1.8600	5.4080	26.6400
P(BS_70_BEPS_30_)	137 ± 8	3.0444	8.9153	29.5840
P(BS_70_NS_30_)	120 ± 10	0.7948	3.0351	13.6101
LDPE *	n.a.	n.a.	4.4079	18.4210

* From Ref. [50].
